# Protein Kinases and Plant Pores

**DOI:** 10.1371/journal.pbio.0040358

**Published:** 2006-10-10

**Authors:** Liza Gross

When water is scarce, plants synthesize a hormone that facilitates conservation by closing stomatal pores on their leaves. Each pore is surrounded by a pair of guard cells that control stomatal aperture in response to various stimuli, including the drought-triggered hormone called abscisic acid (ABA). ABA signaling increases calcium levels in guard cells; calcium in turn acts on a variety of channels that regulate the transport of ions across the cell membranes. As both positively and negatively charged ions (called anions) cross the membrane, turgor pressure drops and stomata close.

These observations support a model in which ABA signaling includes calcium signaling. But ABA signaling also affects parallel pathways and mechanisms—including raising pH levels—and no mutations in calcium-sensing proteins have been previously reported that positively transduce an ABA response in plants. Thus, identifying the molecules that sense and transduce calcium signals in guard cells would provide valuable insights into the mechanics of ABA signaling. Calcium-dependent protein kinases (CDPKs) are calcium-sensor candidates, but with 34 CDPK genes in Arabidopsis alone, functional redundancy in this enzyme family has likely thwarted efforts to characterize their contribution—that is, when the function of one is disrupted, another can step in to fill its role.

In a new study, Izumi Mori, Julian Schroeder, and colleagues describe two CDPK genes—*cpk6* and *cpk3*—with clear roles in calcium and ABA signaling in guard cells. (In previous guard-cell microarray experiments, Schroeder and colleagues had narrowed down the number of guard cell–expressed CDPK genes to a more manageable number.) Losing function of the cpk3 and cpk6 genes in guard cells impairs ABA- and calcium-induces activation of a class of anion channels (slow, or S-type) and stomatal closure. Elevated calcium levels activate S-type anion channels through phosphorylation—a chemical reaction that regulates protein activity; kinases typically function by phosphorylating target proteins.

Plant biologists can investigate gene function by inserting DNA (called transferred DNA, or T-DNA) from the soil bacterium Agrobacterium tumefaciens into a plant's genome. When the inserted T-DNA disrupts a gene's function, researchers can infer gene function based on observed defects in plants carrying the mutant genes, or alleles. After confirming that the *cpk3* and *cpk6* alleles were in fact function-disrupting mutants in Arabidopsis plants, Mori et al. sequenced the alleles and identified two different insertion mutations for both alleles *(cpk3-1* and *cpk3-2*, and *cpk6-1* and *cpk6-2)*. Then they isolated single mutant plants, with two copies of just one allele, and double mutants, with two copies of different combinations of the alleles (for example, two copies of both *cpk3-1* and *cpk6-1* or of *cpk3-2* and *cpk6-2*), for further study.

All the mutant plants looked normal, though both double mutants grew a bit behind schedule compared to the nonmutant (wild-type) plants. ABA-induced stomatal closure, however, was partially impaired. The researchers examined the mutants' effect on calcium and ABA activation of anion channels. In wild-type guard cells, elevated calcium levels activated large S-type anion channel currents, but this activation was significantly reduced in both *cpk3* single mutant cells and even more so in single *cpk6* mutants. Reduced currents were also observed in both double mutants, though they did maintain a background anion current. Double mutants also exhibited reduced ABA activation of the S-type anion channels. Interestingly, Mori et al. also found that ABA activation of another class of ion channels, calcium-permeable channels, was impaired in the single and double *cpk* mutants, revealing the first genetic mutants that impair both ABA regulation of calcium channels and calcium activation of anion channels. Thus, CDPKs play an important role in calcium-mediated ABA regulation of S-type anion channels, calcium channels, and stomatal closing.

This study provides direct genetic evidence that calcium sensors function in stomatal ABA signaling and that CPK3 and CPK6 function as ion channel regulators in guard cell signaling. Because stomatal closing was partially preserved in cells lacking these kinases and another class of “(rapid) R-type” anion channels was less affected in the *cpk* mutants, the authors further conclude that parallel calcium-dependent and -independent signaling mechanisms are at play in a branched guard-cell signaling network. Using a cell-specific signaling and protein regulation approach, as described here, researchers can begin the tall task of characterizing responses of gene-disruption mutants in other members of the large CDPK family function throughout the plant kingdom.

## 

**Figure:**
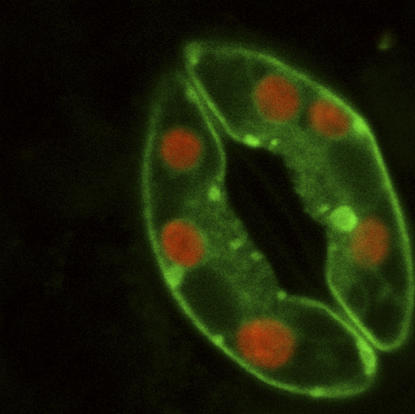
Confocal image of an Arabidopsis stomate showing two guard cells exhibiting green fluorescent protein and native chloroplast (red) fluorescence. (Image: Alex Costa)

